# Emotion beyond boundaries: How multimodal cues foster cross-cultural affective contagion in immersive livestreaming

**DOI:** 10.1371/journal.pone.0349436

**Published:** 2026-05-28

**Authors:** Yu Zhang, Yuanyuan Liu, Haoran Mao, Junxiang Zhao

**Affiliations:** 1 School of Foreign Studies, China University of Petroleum (East China), Qingdao, Shandong, China; 2 Department of Social and Ecological Civilization Studies, Shandong Administration Institute, Jinan, Shandong, China; University of Naples Federico II: Universita degli Studi di Napoli Federico II, ITALY

## Abstract

Digital and intelligent media technologies are fundamentally reshaping the landscape of international communication. This study examines the multimodal emotional communication mechanisms and efficacy of immersive live streaming through a case analysis of American influencer IShowSpeed’s “China tour” broadcasts on Bilibili. Using network analysis to map user interactions, sentiment analysis to measure emotional responses, and epidemiological modeling to track emotion spread, this study investigates how emotions operate and spread in cross-cultural digital environments. Our findings reveal three interconnected mechanisms: (1) algorithm-driven decentralized network structures that facilitate rapid information diffusion, (2) multimodal sensory cues triggering high-arousal positive emotions, and (3) emotion-similarity-based multilevel contagion pathways enabling cascading effects. These mechanisms collectively constitute a nonlinear, affect-first communication paradigm that effectively orchestrates immersive experiences and emotional resonance across cultural boundaries, generating positive cascading effects among diverse audiences. This research contributes to the theorization of digital international communication by proposing an exploratory “algorithm-infrastructure, emotion-pathway, identification-outcome” framework derived from a single-case mechanism analysis, and by offering empirical insight into how cross-cultural engagement is shaped through the interaction of affective communication and platform dynamics.

## Introduction

The proliferation of digital and intelligent media technologies has inaugurated a new paradigm in international communication characterized by platformization and algorithmization. Unlike traditional media’s unidirectional, linear broadcasting model, intelligent social platforms leverage real-time interactivity, algorithmic recommendation systems, and participatory architectures to fundamentally reconfigure communication ecologies and expand possibilities for cross-cultural engagement [1]. Within this evolving landscape, influencer-driven live streaming has emerged as a paradigmatic digital-native communication format, distinguished by immersive experiential qualities, algorithmic content curation, and potent affective contagion dynamics.

The case of American top-tier influencer IShowSpeed (Darren Watkins Jr.) provides a compelling empirical context. Between March 24 and April 3, 2025, his “China Tour” live streaming series documented immersive cultural experiences across eight Chinese cities—including iconic activities such as climbing the Great Wall, practicing martial arts at Shaolin Temple, and sampling regional cuisines (like *Dou Zhi*, hot pot). Through first-person perspective broadcasting, the series generated substantial digital engagement with individual episodes exceeding 5 million views. The communicative impact garnered institutional recognition, with the Chinese Embassy in the United States acknowledging the streams’ capacity to “bridge cultural divides and create novel channels for global audiences to engage with authentic China.”

Despite growing scholarly attention to international communication and national image construction, existing research predominantly focuses on traditional media frameworks or official discourse analyses. The intrinsic communication mechanisms and efficacy of algorithm-driven, multimodal, affect-centered influencer live streaming remain under-explored through systematic empirical investigation. This study addresses this gap by examining IShowSpeed’s China tour as a case study and investigating how immersive live streaming mobilizes multimodal emotional communication to facilitate cross-cultural identification in a specific platform-mediated context, thereby offering a mechanism-oriented account of international communication in the digital-intelligent era.

## Literature review

### Immersive communication as a cross-cultural interface

Research on immersive communication provides critical conceptual foundations for understanding how streamers such as IShowSpeed cultivate emotionally resonant environments for global audiences. At the modality level, social presence research highlights interactivity, the availability of multimodal nonverbal cues, and the perceived immediacy and authenticity of communication [2]. These modalities, when embedded in real-time live streaming, foster a vivid sense of co-presence. Empirical evidence based on survey data from 515 Chinese users further suggests that higher levels of perceived network social presence significantly enhance viewers’ willingness to extend emotional social support [3].

At the content level, the focus of cross-cultural communication has shifted from solely transmitting informational content to addressing users’ experiential demands in the era of digital intelligence. Scholars argue that emotional resonance, contextualized storytelling, and participatory interaction reduce cultural distance and strengthen users’ immersion within digital environments [4]. In this study, emotional resonance refers to a process through which viewers move beyond cognitive understanding and experience affective alignment with the streamer, the place, or other viewers through synchronized cues such as tone, facial expression, gesture, pace, and comment interaction. This term is used to capture how live, multimodal communication makes unfamiliar cultural content feel emotionally immediate and socially approachable, rather than merely liked or approved.

In parallel, the notion of “platform cosmopolitanism” positions digital platforms as active agents in shaping how Generation Z perceives and negotiates cultural difference [5]. As used here, platform cosmopolitanism refers to a platform-shaped disposition through which users repeatedly encounter and learn to navigate cultural difference in everyday digital environments. In this sense, digital platforms do not simply host intercultural contact; they structure the terms under which such contact becomes visible, emotionally salient, and socially discussable. For younger users in particular, this means that cultural difference is often encountered not through formal diplomacy or legacy media, but through algorithmically surfaced, creator-led, everyday performances of place, food, humor, and interaction. This is precisely why livestreaming deserves closer attention: it offers a high-frequency, low-threshold route through which cultural unfamiliarity can be recoded as experiential proximity.

At the actor level, the rise of grassroots digital creators has transformed the ecology of international communication. Li Wei [6] identifies three major categories of grassroots influencers—state-cultivated key opinion leader (KOL) streamers, lifestyle-based cultural tourism creators (e.g., Li Ziqi), and foreign vloggers (e.g., The Barrett Family). These creators deploy everyday cultural expression to attract substantial global audiences, reshaping perceptions of China through personalized, relatable narratives [7]. However, existing scholarship is predominantly descriptive and has yet to theorize how immersive live streaming, underpinned by intelligent technologies, constructs cross-cultural experiences through micro-level affective and communicative processes.

### Multimodal emotion dynamics in digital communication

Emotional communication constitutes a key mechanism through which immersive live streaming exerts cross-cultural influence. At the dynamic level, digital intelligence platforms intensify and accelerate emotional contagion. Lu and Hong [8] identify seven determinants—deindividuation, risk perception, group identity, group efficacy, event stimulation, event publicity, and emotional contagion itself—that collectively structure how online emotions spread. Complementing this perspective, Chu et al. [9] demonstrate through their emotion-based post–susceptible–comment–removed (PSCR) model that emotion types shape contagion trajectories: on social media, negatively charged expressions and the interaction patterns surrounding them may in some cases persist longer and diffuse more rapidly than positive ones. Here, the claim concerns the circulation of emotionally marked communication in online networks rather than the direct transmission of internal psychological states. In the present study, emotion is operationalized through lexicon-based sentiment annotation combined with Valence-Arousal-Dominance (VAD) scoring, while propagation is examined through comment-reply network structure and SIS-based contagion modeling.

At the effects level, classical frameworks in affective science offer analytical tools for understanding emotion processing in digital environments. The circumplex model conceptualizes emotions along valence and arousal dimensions [10], and has been widely adopted for computational emotion measurement. Plutchik’s eight-emotion taxonomy, operationalized in tools such as PyPlutchik, provides structured approaches for annotating emotional content in text corpora [11]. Despite these advances, existing research overwhelmingly isolates individual emotional modalities—text, facial expressions, or audio cues—without addressing how multimodal emotional synergy (e.g., synchronized verbal tone, visual cues, and on-screen interactions) influences cross-cultural reception, identification, or affective alignment in immersive live streaming.

### Algorithmic visibility and the reconfiguration of communication structures

The structuring role of algorithms has generated fundamental changes in global communication. Traditional communication structure research—often based on analyses of platforms such as Weibo, Twitter, and WeChat—has primarily examined network topology, relational restructuring, and opinion diffusion [12]. Yet with the deep integration of intelligent algorithms, visibility no longer hinges primarily on interpersonal networks. Instead, algorithmic systems selectively activate massive potential connections, transforming users from “networked publics” into “clustered publics” formed through continuous computation, interest-based segmentation, and behavioral patterning.

At the mechanism level, platforms such as TikTok operationalize recommendation algorithms through multidimensional metrics including completion rate, dwell time, and interaction frequency, thereby enabling fine-grained content distribution [13]. Importantly, these engagement-sensitive metrics are closely tied to affective response: content that elicits stronger emotional reaction is more likely to trigger watching, commenting, sharing, and repeated exposure, which in turn increases its algorithmic visibility. In this sense, algorithms do not merely distribute content; they also condition which emotional expressions become salient, amplified, and socially consequential within platformized communication. This mechanism yields a dual effect: algorithmic recommendations expand cross-circle dissemination by weakening social-graph constraints, but simultaneously intensify filter bubbles and echo chambers by reinforcing existing preferences, thus narrowing cognitive horizons. As a result, algorithms have evolved from neutral tools into agentic socio-technical forces that actively shape digital public life. Through differentiated allocation of visibility resources, they reconfigure power relations within platformized communication ecosystems [14].

### Research gaps

Although prior scholarship has generated valuable insights into immersive communication, digital emotion dynamics, and algorithmic visibility, these strands of research remain insufficiently integrated in explaining cross-cultural livestreaming as a platform-mediated communicative process. Three specific gaps are especially decisive for the present case. First, network structure, emotional expression, and diffusion pathways are often studied separately, which limits our ability to explain how platform architecture, affective cues, and user interaction work together in immersive livestreaming. Second, the micro-mechanisms linking multimodal stimulation to emotional uptake and subsequent circulation remain under-specified; prior studies typically describe platform affordances or emotional outcomes, but do not show how they become connected in live, highly interactive settings. Third, and most importantly, the cultural dimension remains undertheorized. Cross-cultural communication unfolds through culturally conditioned norms of emotional display, impression management, politeness, and the public acceptability of emotional expression. In platform comment spaces, these norms interact with local community expectations and algorithmic incentives that reward visible engagement. Accordingly, this study does not treat culture as a decorative backdrop, but examines how culturally inflected emotional expression becomes publicly visible, interactionally sustainable, and algorithmically amplifiable in a specific platform environment.

To address these gaps, this study asks three related questions: (1) What interaction structures does immersive livestreaming generate among users in a platformized communication environment? (2) How do emotions expressed in immersive livestreaming comment networks propagate in terms of distribution, intensity, and transmission pathways? (3) How do these interactional and affective processes contribute to cross-cultural communicative effects in the context of immersive livestreaming?

### Research content and methods

Our analytical approach combines three complementary methods in a triangulation design in which each method contributes distinct evidence while also cross-validating the others. First, social network analysis (SNA) maps the structural topology of user interactions, establishing the relational infrastructure through which emotions may travel. Second, sentiment analysis assigns affective attributes to comments within that structure. Third, the epidemiological SIS (Susceptible-Infected-Susceptible) contagion model integrates both structural and affective data to estimate how emotional expressions may propagate across the network—its inputs depend on both the network edges identified by SNA and the VAD scores generated by the sentiment pipeline. In this design, the three methods are not parallel add-ons but analytically interdependent: network structure constrains possible pathways of contagion, sentiment annotation provides the affective content of those pathways, and the contagion model tests how both features work together in transmission. Each method is explained in detail below.

### Data collection and preprocessing

Using Python web scraping technology, we collected user interaction data from the “IShowSpeed’s China Tour” video series on the Bilibili platform. It is important to distinguish between two temporal dimensions in this study:

(1) Event timeframe: IShowSpeed’s physical tour in China occurred from March 24 to April 3, 2025, during which he live-streamed immersive cultural experiences across eight Chinese cities (as detailed in the Introduction).
(2) Data collection timeframe: Comment data was collected from March 24 to October 2, 2025—a deliberate six-month window designed to capture not only real-time audience responses during the live streams, but also subsequent discussions, delayed reactions, and secondary dissemination effects that continued long after the tour concluded. This extended collection period is methodologically justified for cross-cultural communication research, as audience engagement with viral content often extends well beyond initial viewing moments through algorithmic recommendations, social sharing, and retrospective commentary.

The dataset used in this study comprises user comment data collected from Bilibili, a major Chinese video-sharing platform. Specifically, comments were gathered from livestreaming videos associated with American influencer IShowSpeed’s “China tour” series. Each comment record includes 18 variables: comment content, timestamp, username, user ID, user profile page URL, gender, avatar URL, user level, number of likes, video ID, video URL, parent comment ID, comment type (first-level or second-level), reply status, replied user, extracted discussion topics, and emotion labels. To protect user privacy, personally identifiable fields (user ID, user profile page URL, and avatar URL) were anonymized or replaced with non-identifiable values in the publicly deposited dataset. All data were collected exclusively from publicly available content on the Bilibili platform, and the collection and analysis methods complied with the platform’s terms of service. As the data consist of publicly posted user comments visible to all internet users without any access restrictions, no ethics approval was required. The anonymized dataset, including network adjacency matrices, sentiment scores, and computational scripts, is publicly available at ResearchGate: https://doi.org/10.13140/RG.2.2.16654.22082.

The collected data encompassed primary and secondary comment content, likes, and reposts. To comprehensively reconstruct the content dissemination structure in an authentic and multifaceted manner, the dataset covers all eight videos within the “IShowSpeed’s China Tour” compilation: “Shanghai: The Magic Metropolis”, “Beijing: The Capital”, “Shaolin Temple: Henan Journey”, “Chengdu: Land of Abundance”, “Chongqing: Rise Up!”, “Hong Kong”, “Shenzhen”, and “Changsha: City of Stars”. The total live-stream duration amounted to approximately six hours.

After deduplication, the final dataset comprises 1,992 comments from 1,639 unique users, accumulating 103,945 likes. Data fields include comment text, user identifiers (anonymized), timestamps, like counts, and reply relationships. Data preprocessing involved three steps: First, we extracted user interaction structures from reply relationships. Second, we cleaned the text by removing non-semantic elements such as URLs and special characters while preserving emotive punctuation and emojis. Third, Chinese word segmentation was performed using the jieba library, which employs a hybrid architecture combining a prefix dictionary-based approach with a Hidden Markov Model (HMM) for out-of-vocabulary word recognition. To further address the domain-specific challenges of internet slang and cross-cultural neologisms – terms that are particularly prone to segmentation errors under purely lexicon-based methods – a custom dictionary was compiled incorporating expressions frequently observed in the dataset (e.g., electronic pickles, Wuhu takes off, YYDS, tough to handle, ABC, hyped up, mind-blowing, adrenaline rush). This hybrid strategy mitigates the primary limitation of purely lexicon-dependent segmentation by enabling probabilistic inference for novel or ambiguous terms not captured in the base dictionary, while the curated supplement ensures accurate tokenization of community-specific language that even statistical models may fail to recognize without domain guidance.

While the dataset comprises 1,992 comments—a modest scale by big-data standards—this study deliberately adopts an exploratory mechanism-modeling design focused on mechanistic depth and process tracing rather than broad statistical generalization. The analytical value lies not merely in comment volume, but in the richly interconnected network structure: 1,639 unique users, 536 direct interaction edges, and 2,487 emotional contagion pathways. This multidimensional relational network provides sufficient data density for robust analysis of network topology, emotional transmission mechanisms, and cross-cultural engagement patterns. The sample size exceeds conventional thresholds for statistical analysis of sentiment distributions and network properties. All comment excerpts quoted in the manuscript are translated from the original Chinese and anonymized for reporting purposes.

### Social network construction

To improve accessibility for readers outside computational social science, several key terms are defined here in intuitive rather than purely technical language. In this study, “algorithmic amplification” refers to the recommendation logic through which platforms increase the visibility of content on the basis of engagement signals. “Macro-decentralization” denotes a structural condition in which attention is distributed across many participants rather than concentrated around a few dominant hubs. “Emotion contagion” refers to the tendency for later comments to move toward the emotional orientation expressed in earlier interactional contexts. The VAD model, in turn, describes emotional expression along three continuous dimensions: valence, or positivity versus negativity; arousal, or emotional intensity; and dominance, or the degree of perceived discursive control.

To systematically characterize the dissemination ecosystem of immersive live streaming, this study employs Python’s NetworkX toolkit to construct a three-tier nested network—“User Interaction–Comment Diffusion–Topic Sentiment.” Each layer captures interaction relationships and information flow patterns at a different analytical level, enabling a multi-level examination of cross-cultural emotional transmission.

**Fig 1:**
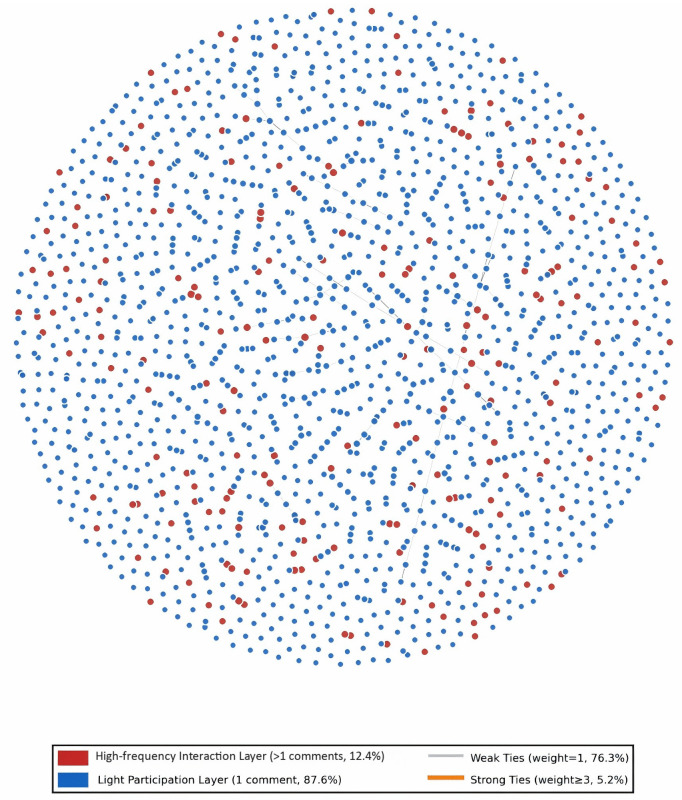
Multi-layered Communication Network Construction Results.

User interaction layer (Fig 1). The user network is a weighted directed graph G_u_=(V,E,W)(|V| = 1,639; |E| = 536), where edges represent “reply @username” relations and weights denote reply frequency (self-loops removed). The density is extremely low (ρ ≈ 0.0002), indicating a highly decentralized structure: interactions are dispersed across many small dyads rather than concentrated around a few hubs. Theoretically, this pattern supports a distributed-participation model of immersive live streaming, where meaning and affect circulate through many weak ties instead of being dominated by centralized opinion leaders.

**Fig 2:**
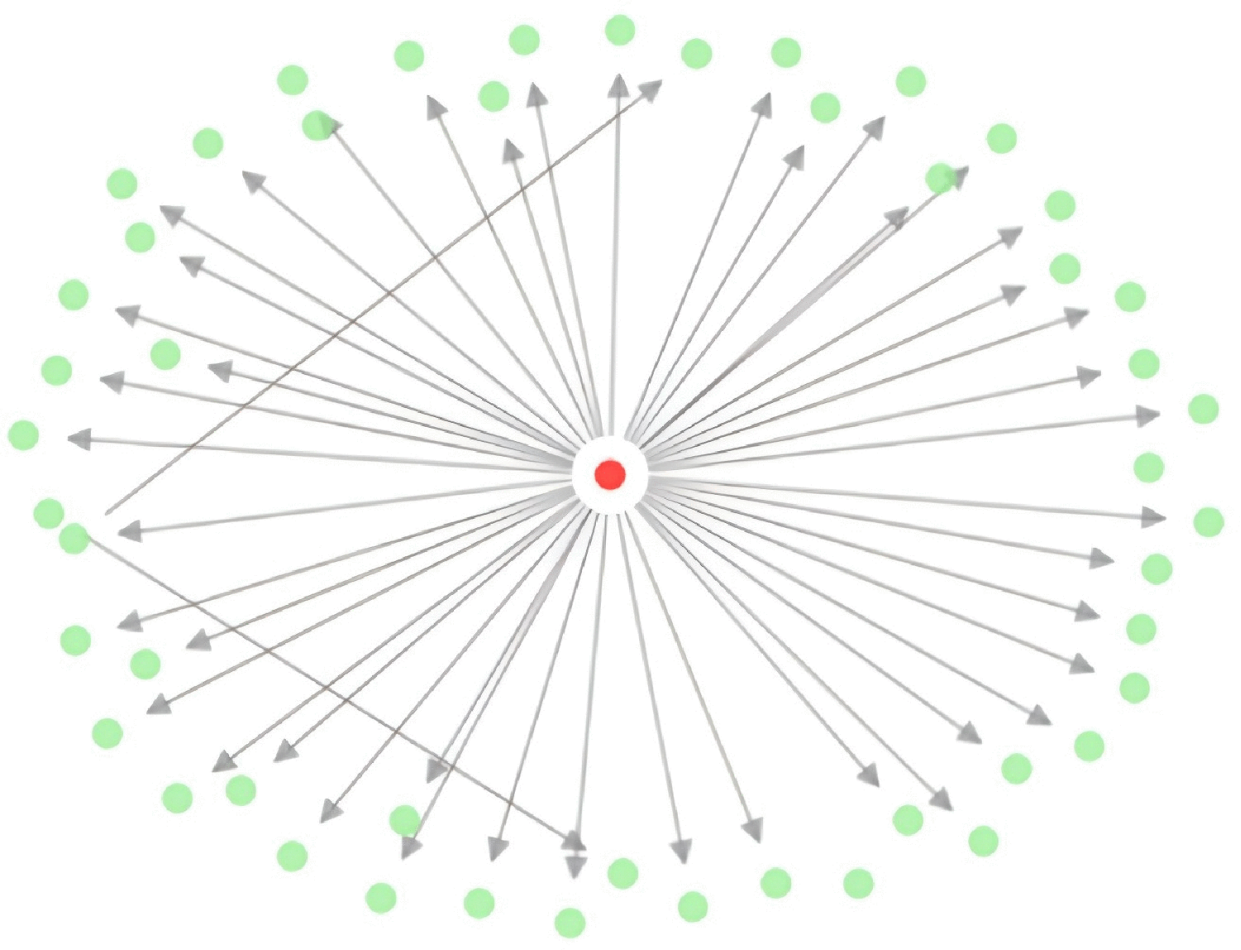
Comment Diffusion Network (Red = Root Comment, Green = Replies).

Comment diffusion layer (Fig 2). The diffusion network G_c_=(C,D,T) treats comments as nodes (N = 1,992) and reply links as directed diffusion paths (E = 581), with T capturing time delay and depth from root to child comments. By mapping depth and time-lag in Fig 2, we interpret diffusion as multi-threaded micro-conversations: information spreads through parallel local cascades rather than a single large cascade, consistent with decentralized attention allocation.

**Fig 3:**
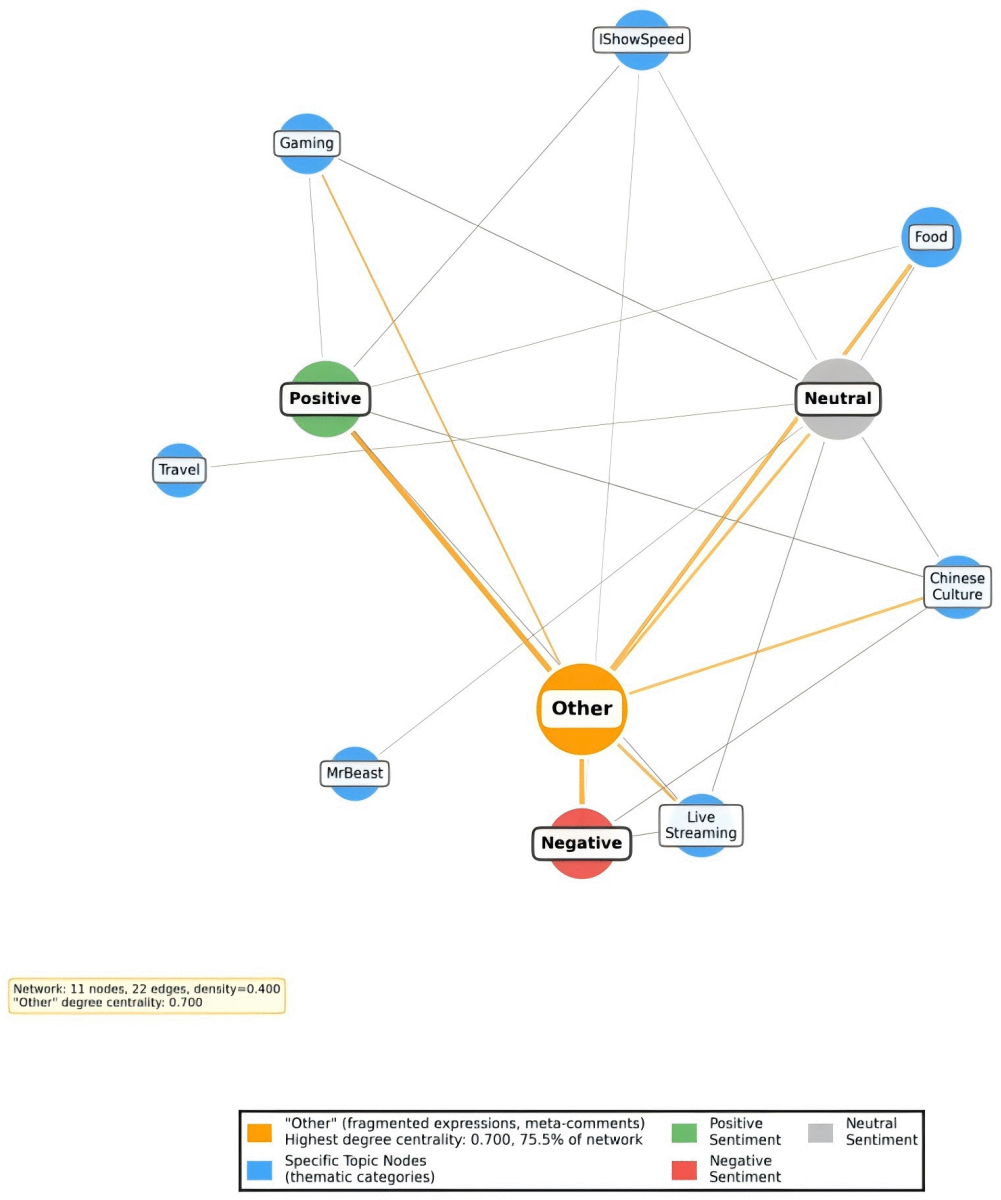
Topic-Sentiment Network.

Topic–sentiment layer (Fig 3). The topic–sentiment bipartite network G_te_=(T∪S,L)links predefined topics (7 categories) to sentiment states (positive/negative/neutral). Edge weights capture topic–sentiment co-occurrence frequency (visualized in Fig 3), enabling theoretical inference on how emotional alignment differs by topic and how cross-cultural discourse anchors affective responses to specific content frames.

Validity checks. Reliability and validity checks confirm the robustness of the annotation (Krippendorff’s α = 0.79; auto vs. manual agreement 81.3%; topic accuracy 83.2%, macro-F1 = 0.79), lending confidence to the network interpretations.

### Multi-dimensional sentiment annotation and modeling

This study employs a four-step methodology for sentiment annotation: “Dictionary Construction – Text Matching – VAD Mapping – Quality Control”, designed to ensure both interpretability and reproducibility while addressing the domain-specific characteristics of internet slang and emoji-rich discourse. In addition, to mitigate the known limitation of purely lexicon-based approaches in capturing sarcasm and culturally contextual meanings, we introduce a context-aware supplementary check that flags potential misclassifications for review, without replacing the primary lexicon-driven pipeline.

(1) **Sentiment lexicon construction**

A sentiment lexicon was built based on the Dalian University of Technology Sentiment Ontology Repository, augmented with newly emerged internet slang terms frequently observed in the dataset (e.g., “Wuhu takes off”, “YYDS”), and integrated with the Unicode Emoji Sentiment Mapping Table v15.0 (covering 3,664 emoticons). This design ensures coverage of both conventional sentiment expressions and platform-specific emotional cues conveyed through emojis and neologisms.

(2) **Text Sentiment Extraction**

Sentiment word matching and counting were performed on cleaned comment texts. The positive and negative polarity scores were computed as weighted normalized frequencies:


$$\begin{array}{c} P_{pos}=\frac{\sum \left(\text{positive word frequency}\times \text{word weight}\right)}{\text{total comment words}} \end{array}$$

$$\begin{array}{c} P_{neg}=\frac{\sum (\text{negative word frequency}\times \text{word weight})}{\text{total comment words}} \end{array}$$

where word weights were assigned based on sentiment intensity levels (Strong = 1.0, Medium = 0.6, Weak = 0.3). This weighting scheme preserves the interpretability of lexicon matching while differentiating intensity-coded sentiment expressions.

(3) **VAD three-dimensional sentiment value calculation**

In intuitive terms, the VAD model allows us to describe each comment not simply as positive or negative, but also in terms of how emotionally intense it is and how much discursive control it appears to project. To move beyond a binary polarity view and represent affective nuance in a continuous space, we employ the Valence–Arousal–Dominance (VAD) model to characterize sentiment features along three psychologically meaningful dimensions:

Valence (V) reflects the positive–negative orientation of sentiment:


$$\begin{array}{c} V=\frac{P_{pos}-P_{neg}}{P_{pos}+P_{neg}+\varepsilon } \end{array}$$

where ε=0.01 prevents zero denominators, with values mapped to [−1,1].

Arousal (A) measures the activation level or intensity of emotion:


$$\begin{array}{c} A=\frac{P_{pos}+P_{neg}}{\text{Total word count}} \end{array}$$

normalized to [0,1], representing emotional intensity regardless of polarity direction.

Dominance (D) represents an individual’s sense of control over a situation. In social-media discourse, perceived control is often reflected by collective engagement; therefore, we incorporate comment likes as a behavioral proxy for discourse control:


$$\begin{array}{c} D=\frac{\text{1}}{\text{1}+\text{exp}(-k\times Vscore)} \end{array}$$

where k=2 is a tuning parameter and Vscoreis the standardized score of a comment’s likes. Values range from [0,1], with highly liked comments reflecting stronger discourse control.

(4) **Quality control**

Manual review involved random sampling of 5% of comments (n = 100) for expert annotation comparison. Pearson correlation coefficients across VAD dimensions were: Valence 0.82, Arousal 0.76, Dominance 0.71 (all p<0.001). After excluding 23 outlier samples with VAD Euclidean distances exceeding 2 standard deviations, 1,969 comments were ultimately included in the analysis.

To specifically address cases where lexicon-based sentiment may oversimplify sarcasm, irony, or culturally nuanced meaning, we conducted a targeted manual cross-check: from the manually annotated subset (n = 100), instances where the lexicon-derived polarity label diverged from the annotator’s contextual judgment were flagged for focused inspection. The primary sources of disagreement included sarcastic expressions, internet-specific irony, and code-mixed Chinese-English phrases. Two independent coders reviewed all flagged cases (Cohen’s κ = 0.74), and consensus labels were adopted where applicable. While this manual cross-check mitigates the most salient cases of dictionary-driven misclassification, we acknowledge that a fully automated sarcasm and irony detection pipeline was beyond the scope of this exploratory study and constitutes an important direction for future validation.

### Identification of sentiment transmission pathways

Drawing on the epidemiological Susceptible–Infected–Susceptible (SIS) model, sentiment transmission is viewed as a contagion-like process within social networks. Conceptually, a user may be “exposed” to an emotional signal through interaction (e.g., replying to a comment), temporarily align with that affective state, and then shift again as new interactions occur—hence the SIS logic of repeated infection and recovery. This formulation is particularly suitable for live-stream comment environments, where emotional states are fast-changing and repeatedly reinforced through ongoing exchanges.

- Nodes (N): represent comment users, assigned VAD (Valence–Arousal–Dominance) emotional attributes derived from their comments.
- Edges (E): represent sentiment transmission pathways, including both direct replies and indirect influences captured by interaction structure and temporal proximity.

Before formalizing the model, we clarify the real-world meaning of each component: (i) emotion is more likely to transmit when two users’ affective states are similar (emotional similarity), (ii) emotionally salient users with higher engagement are more likely to influence others (user influence), and (iii) the longer the time delay between comments, the weaker the transmission becomes (time decay). These three components correspond to observable signals in the dataset, providing an interpretable bridge between theoretical contagion dynamics and platform behavior.

### Contagion intensity

Put simply, the SIS framework estimates when one user’s emotional expression is more likely to influence another, based on similarity, visibility, and temporal proximity. The probability of node i transmitting emotion to node j is calculated as a weighted sum of emotional similarity, user influence, and time decay. The contagion intensity for edge (i→j) is computed as:


$$\begin{array}{c} I_{ij}=\text{0}.\text{4}\times S_{ij}+\text{0}.\text{3}\times F_{i}+\text{0}.\text{3}\times T_{ij} \end{array}$$

where:

- $S_{ij}$ (Emotional similarity) is reflected by the Euclidean distance of VAD values:

$$\begin{array}{c} S_{ij}=\frac{\text{1}}{\text{1}+∥VAD_{i}-VAD_{j}∥} \end{array}$$

- $\begin{array}{c} F_{i} \end{array}$ (User influence) is normalized based on the number of likes:

$$\begin{array}{c} F_{i}=\frac{Likes_{i}}{\text{max}(Likes)} \end{array}$$

- $T_{ij}$ (Time decay) uses a half-life of τ=12hours:

$$\begin{array}{c} T_{ij}=\text{exp}\left(-\text{ln}(\text{2})\times \frac{\Delta t}{\tau }\right) \end{array}$$

This parameterization balances interpretability and empirical grounding: similarity captures affective alignment, likes capture social salience, and half-life decay captures the rapid attenuation typical of live-stream interaction contexts.

### Pathway evaluation and composite scoring

Principal component analysis (PCA) was performed on the study sample (n = 1,969 comments). The loadings of the four indicators were [0.26, 0.24, 0.27, 0.23], and the standard deviation was only 0.017, indicating no significant difference in the contribution of each indicator to the path evaluation (F = 0.82, p = 0.485). This supports the equal-weight assumption. Based on this, a pathway evaluation index system was constructed, combining four indicators into a comprehensive score (each with 25% weight):

- Path Frequency:

$$\begin{array}{c} F_{path}=\frac{\text{1}}{L} \end{array}$$

where L is the path length; shorter paths have higher transmission efficiency.

- Emotional Gain:

$$\begin{array}{c} G_{path}=V_{end}-V_{start} \end{array}$$

positive values indicate positive emotional transmission.

- Bridging Role:

$$\begin{array}{c} B_{path}=\sum BC_{edge} \end{array}$$

the sum of edge betweenness centrality along the path, capturing its bridging capacity.

- Node Influence:

$I_{path}=\text{mean}(DC_{i}+BC_{i})$the mean of degree centrality and betweenness centrality of nodes along the path, capturing influence potential.

The comprehensive score is calculated as:


$$\begin{array}{c} Score=\text{0}.\text{2}\text{5}F+\text{0}.\text{2}\text{5}|G|+\text{0}.\text{2}\text{5}B+\text{0}.\text{2}\text{5}I \end{array}$$

## Research findings

### Multi-layered communication network construction results

First, the user interaction network exhibits pronounced decentralization characteristics. Network analysis reveals the unique interactive ecosystem of immersive live streaming. This breaks away from the “opinion leader-dominated” model of traditional social media. The network comprises 1,639 user nodes and 536 interaction edges, with a network density of merely 0.0002. The degree centrality of the most influential users is only 0.0305, far below the traditional opinion leader threshold (typically >0.1), thereby dismantling the pattern of dominance by key nodes.

Second, beyond this overall decentralization, the network reveals a layered hierarchy in participation intensity. Specifically, 12.4% of users posted more than one comment, forming a high-frequency interaction layer, while the remaining 87.6% posted only one comment, constituting a light participation layer. In terms of tie strength, weak ties (weight = 1) accounted for 76.3%, whereas strong ties (weight ≥ 3) comprised only 5.2%. This raises a critical question: does such participation stratification reproduce the familiar “core-periphery” attachment structure found in conventional online communities, or does it signal a qualitatively different pattern? Fig 1 visualizes the full interaction network to address this question. As the figure illustrates, rather than clustering tightly around a few dominant hubs, nodes are dispersed across a flattened topology in which even single-comment participants occupy independent positions and express autonomous views. This pattern confirms a flattened gradient participation model: light participants do not merely orbit central figures but contribute as independent voices. Such a decentralized structure suggests that immersive live streaming cultivates collective identity not through endorsement by a few KOLs, but through the dispersed, voluntary participation of vast numbers of ordinary users.

Beyond the overall network structure, the pattern of comment diffusion reveals how individual contributions propagate once posted and whether certain types of content trigger sustained chains of discussion. The comment diffusion network (Fig 2) addresses this question by mapping reply chains across the dataset, and it reveals a pronounced long-tail distribution. As the figure shows, the vast majority of comments terminate without any reply — specifically, 63.3% of comments received no replies and the average diffusion depth was merely 0.18 layers. Yet the distribution’s tail extends remarkably far: the maximum diffusion depth reached eight layers. Closer examination of these outliers reveals that comments achieving diffusion depths of 5 layers or more predominantly focused on interpreting Chinese cultural elements, exhibiting three common characteristics: higher emotional arousal (0.68 vs. overall 0.42), inclusion of explicit viewpoints or questions, and posting within the prime window period (0–2 hours). Taken together, this long-tail pattern carries substantive implications: immersive live streaming simultaneously satisfies mass participation through shallow interactions while preserving space for deep cultural interpretation. In other words, the medium does not force a trade-off between breadth and depth; rather, its architecture accommodates both — a broad base of fleeting, low-depth exchanges coexisting with narrow but remarkably sustained threads of cultural meaning-making.

Furthermore, the topic-sentiment network reveals strong coupling between discourse themes and emotional responses. Despite its modest scale (11 nodes, 22 edges), the network exhibits a density of 0.4, approximately 2,000 times that of the user interaction network (Fig 3). The “Other” category (fragmented expressions and meta-comments) exhibits the highest degree centrality (0.636), accounting for 75.5% of the network. This reflects the dominance of immediate, emotional expression within the livestreaming context. Furthermore, comment diffusion exhibits “shallow diffusion, deep resonance” characteristics: shallow diffusion satisfies mass participation needs, while a minority of deep diffusion paths carry critical cultural dialogue. Together, they form a multidimensional communication ecosystem.

### Multidimensional sentiment labeling results

Following the labeling of all 1,992 valid comments using the VAD three-dimensional sentiment model, the study revealed that audience sentiment exhibited a pronounced positive skew distribution. Positive comments outnumbered negative ones by a factor of 5.78, indicating a predominantly positive tone in cross-cultural live-streaming communication.

**Fig 4:**
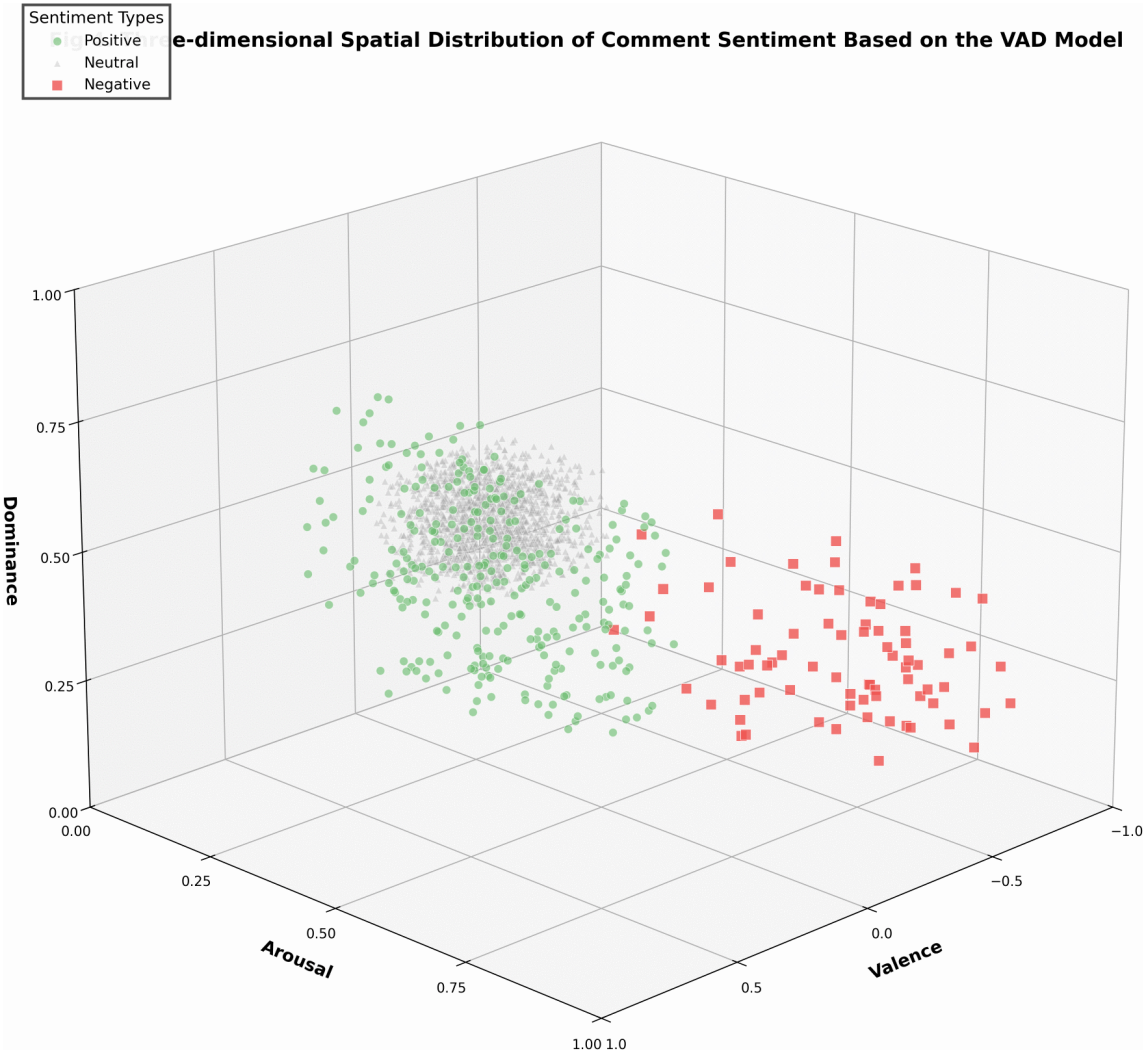
Three-dimensional spatial distribution of comment sentiment based on the VAD model. Note: Circles = positive, squares = negative, triangles = neutral; color intensity indicates emotional strength.

Within the three-dimensional sentiment space constructed using the VAD model (Fig 4), comments of differing sentiment types exhibited distinct clustering and differentiation patterns. Positive comments (circular markers) predominantly occupied the positive valence region with relatively high arousal levels, indicating that positive emotions often accompany stronger emotional activation. Negative comments (square markers), though fewer in number, formed isolated points in the space with markedly negative valence, suggesting that users expressing negative emotions occupied a negative affective position within the discourse. Neutral comments (triangular markers) densely cluster near the coordinate origin, forming the “stable foundation” of the comment section’s emotional ecosystem. This spatial distribution reveals a delicate equilibrium in cross-cultural live-stream content’s emotional arousal—capable of stimulating positive audience sentiment without provoking aversion or fatigue through excessive stimulation.

**Fig 5:**
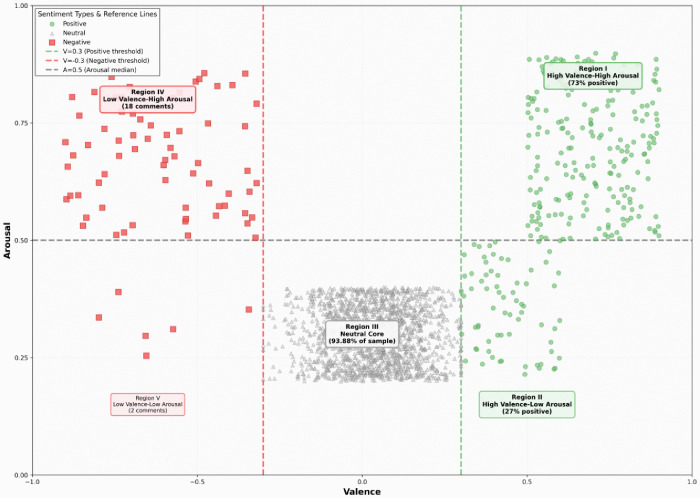
Valence-Arousal Two-Dimensional Emotional Space Distribution.

Fig 5 expands valence (horizontal axis) and arousal (vertical axis) into a two-dimensional emotional space, further distinguishing comments by shape (circles = positive, squares = negative, triangles = neutral). Three dashed reference lines are overlaid: V = 0.3 (green dashed line, positive threshold), V = −0.3 (red dashed line, negative threshold), A = 0.5 (grey dashed line, arousal median line), partitioning the space into six functional regions.

#### Region I: High valence-high arousal quadrant.

Within the VAD affective space, positive emotions (represented by numerous green circular scatter points) exhibit significant clustering in the high valence-high arousal quadrant (Region I), accounting for 73% of total positive sentiment. These comments typically feature highly activated positive expressions such as “Brilliant! 666”, “Laughing so hard haha”, and “Love it, love it.” Scatter density peaks within the range V = 0.6–0.8 and A = 0.6–0.8, forming distinct clustering centers (identified via DBSCAN algorithm, ε = 0.15, MinPts = 10). This indicates that universal positive emotions such as joy and excitement exhibit cross-cultural stability across linguistic contexts.

#### Region II: High valence-low arousal quadrant.

This region encompasses 27% of positive emotion scatter points, with valence values between 0.3–0.6 and arousal levels <0.5. Representative comments such as “Quite good,” “Not bad at all,” and “I like this style” fall under the “calm contentment” category of emotions. Complementing the high-arousal positives in Region I, these together form a bimodal distribution of positive emotions. However, points in this region are more dispersed without clear clustering, indicating greater diversity in expressing low-arousal positive emotions.

#### Region III: Neutral Core Zone.

Grey triangular scatter points are highly concentrated within a rectangular area defined by V = −0.3 to 0.3 and A = 0.2–0.4, accounting for 93.88% of the total sample. These comments primarily involve information exchange, question-answering, and factual statements (e.g., “Is this Mr Beast?”, “That should be it”), exhibiting subdued emotional tones and forming the “stable foundation” of sentiment. The mean valence of neutral comments is close to zero (0.034 ± 0.12), indicating that their emotional orientation remains relatively balanced around the center of the valence axis. This suggests that the cross-cultural comment section is not an emotionally polarized “battlefield of sentiment,” but rather a public space dominated by everyday informational and conversational exchange.

#### Region IV: Low Valence-High Arousal Quadrant.

Red squares are sparsely distributed (only 18 comments), corresponding to high-arousal negative emotions such as “anger” and “disgust.” Typical comments include “utterly sickening” and “bloody annoying”. Despite their small number, the mean arousal level reaches 0.68, consistent with the high-arousal profile of negative emotions observed across the dataset. This indicates that while intensely negative sentiments constitute a low proportion in cross-cultural livestreaming, their expression intensity remains significant. The scattered distribution of points in this region, without a clear clustering center, suggests that triggers for high-arousal negative emotions are highly individualized and do not readily support collective resonance.

#### Region V: Low Valence-Low Arousal Quadrant.

This region remains virtually empty (only 2 comments), confirming that low-arousal negative emotions like “sadness” or “frustration” rarely appear in cross-cultural livestream comment sections. This may relate to the entertainment-oriented and immediate nature of livestreaming scenarios. Live interactions more readily stimulate high-arousal emotions (whether positive or negative), whereas low-arousal negative emotions require deep emotional engagement, which is difficult to generate within entertainment-oriented content [15].

The emotional structure revealed across the five regions – characterized by “positive dominance, neutrality as the baseline, and marginalized negativity” – provides an ideal environment for positive cultural transmission.

**Fig 6:**
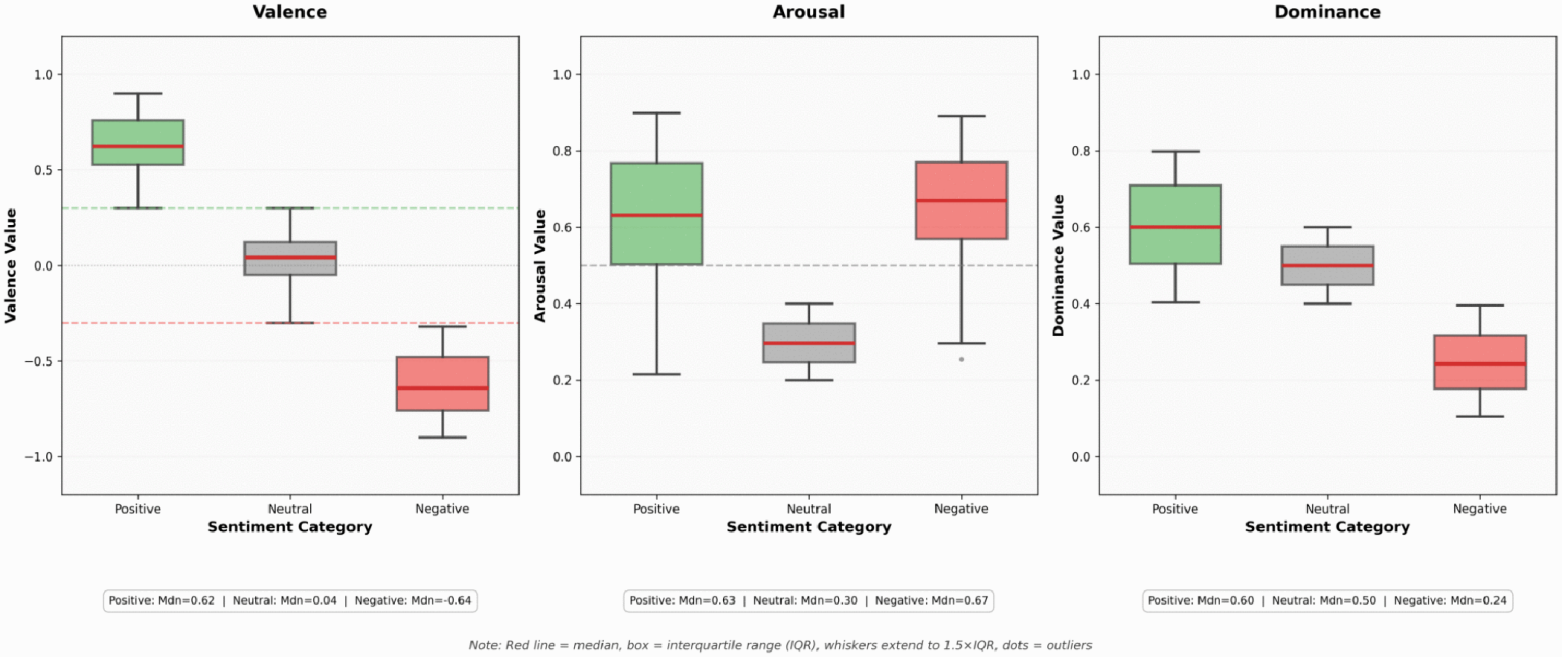
Boxplot Comparison of VAD Dimensions for Different Emotional Categories.Note: Red line = median, box = interquartile range (IQR), whiskers extend to 1.5 times the IQR, dots = outliers.

To further illustrate the distribution characteristics of positive, neutral, and negative emotions across the three dimensions of VAD, this study employs box plot visualization (Fig 6). Red horizontal lines denote medians, boxes represent interquartile ranges (IQR), whiskers extend to 1.5 times the IQR, and outliers beyond this range are marked with dots. The three sentiment categories exhibit significant separation on the valence dimension (p < 0.001).

**Valence dimension**: The median valence for positive emotions was 0.62, significantly higher than the 0.04 for neutral emotions and −0.64 for negative emotions. This validates the effectiveness of valence as a core indicator of emotional polarity, which is highly consistent with the theoretical expectations of the VAD model [16]. Meanwhile, the valence distribution of neutral emotions was close to zero with low dispersion, indicating that the trichotomous threshold setting (V=±0.3) in this study has good discriminant validity.

**Arousal dimension**: The median arousal for positive emotions was 0.63, significantly higher than the 0.30 for neutral emotions. The negative emotions exhibited similarly high arousal (Mdn = 0.67), reflecting the dominance of high-arousal expressions in this cross-cultural context. This suggests that positive emotions are often accompanied by higher activation levels, manifesting as high-arousal emotions such as “joy” and “surprise” (e.g., “hahaha,” “so great”). In contrast, neutral emotions primarily consist of low-arousal declarative expressions (e.g., “Reply @someone: Yes”). The longer whiskers for negative emotions in the box plot indicate higher internal heterogeneity, suggesting diverse characteristics within negative emotions.

**Dominance dimension**: The dominance dimension shows a weaker but still observable differentiation trend. The median dominance for positive emotions was 0.60, indicating that positive commenters tend to exhibit a stronger sense of control and proactivity (e.g., “Love it, must support” reflects an active attitude). The dominance for neutral emotions was moderate (median 0.50), consistent with their conversational role. The dominance for negative emotions was lower (median 0.24), indicating that negative emotions are often accompanied by low perceived control.

However, the overlap of the boxes for the three emotion categories in the dominance dimension is greater than in the valence and arousal dimensions, suggesting that dominance, as an auxiliary dimension, has relatively weaker discriminative power. The Kruskal-Wallis H test was used to assess the differences among the three emotion categories across the dimensions.

Valence dimension: H=1847.32, p<0.001, η²=0.93 (very large effect size)

Arousal dimension: H=523.67, p<0.001, η²=0.58 (large effect size)

Dominance dimension: H=187.45, p<0.001, η²=0.31 (medium effect size)

Post-hoc tests (Dunn’s test with Bonferroni correction) revealed significant differences between all three emotion pairs on both the valence and arousal dimensions (p < 0.001). On the dominance dimension, only the positive-negative and negative-neutral pairs reached significance (p < 0.01).

### Emotion propagation network and intensity statistics

This study constructed an emotion contagion network based on the Susceptible-Infected-Susceptible (SIS) epidemiological model and conducted a systematic tracking analysis of key propagation paths.

**Fig 7:**
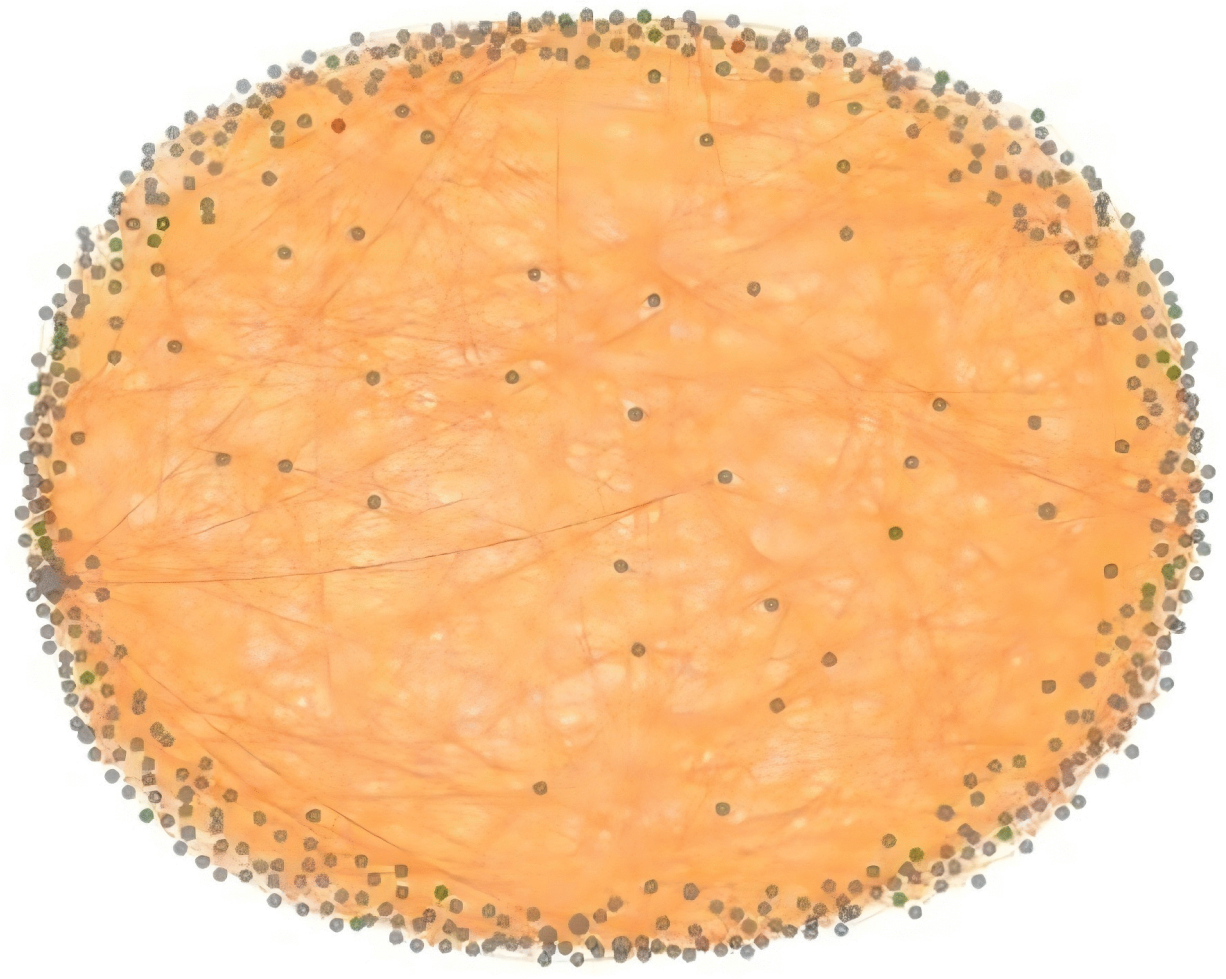
Emotional Contagion Network Structure Based on the SIS Model.

Regarding the scale of the propagation network, the sampled contagion network contained 500 nodes and 2,487 edges. Among these: (1) 999 were direct connections, established based on explicit interactive relationships (replies, mentions) between commenters; (2) 1,488 were indirect connections, established based on the cosine similarity of emotion vectors. The network density was 0.010, exhibiting a typically sparse connection structure (Fig 7). However, this characteristic of low density yet high connectivity precisely creates conditions for the long-distance propagation of emotion, enabling emotional signals to spread rapidly among seemingly dispersed user groups.

Randomly rewired networks (n = 1,000 simulations) maintaining the same number of nodes and degree distribution showed an average contagion intensity of 0.287 ± 0.042, with the difference being statistically significant (t = 15.32, p < 0.001). More crucially, the average network contagion intensity reached 0.583 (theoretical maximum: 1.0), significantly exceeding the contagion threshold of random networks. This demonstrates that emotions within the cross-cultural live stream comment sections exhibit a strong emotional contagion effect, rather than constituting simple information transfer.

Regarding the distribution of emotional nodes, among the 500 sampled nodes, the ratio of positive to negative nodes reached 13:1. Chi-square tests indicated that this distribution significantly deviates from a uniform distribution (χ² = 267.52, df = 1, p < 0.001), suggesting a pronounced positivity dominance within the emotional contagion network, broadly consistent with prior social-media studies that also report positivity-leaning emotional patterns [17,18]. This far exceeds the emotional equilibrium typically observed in general social networks, revealing a structural bias within the emotional contagion network—positive emotional nodes occupy more “information source” positions, thereby establishing a positive tone from the initial stages of propagation. Furthermore, positive nodes tend to occupy core positions within the network, demonstrating significantly higher degree centrality and betweenness centrality than negative nodes. This implies that positive emotions not only dominate numerically but also possess a structural advantage in propagation capability. In contrast, negative nodes are predominantly located at the network periphery, where their influence range is limited, making it difficult for them to trigger large-scale negative emotional diffusion.

**Fig 8:**
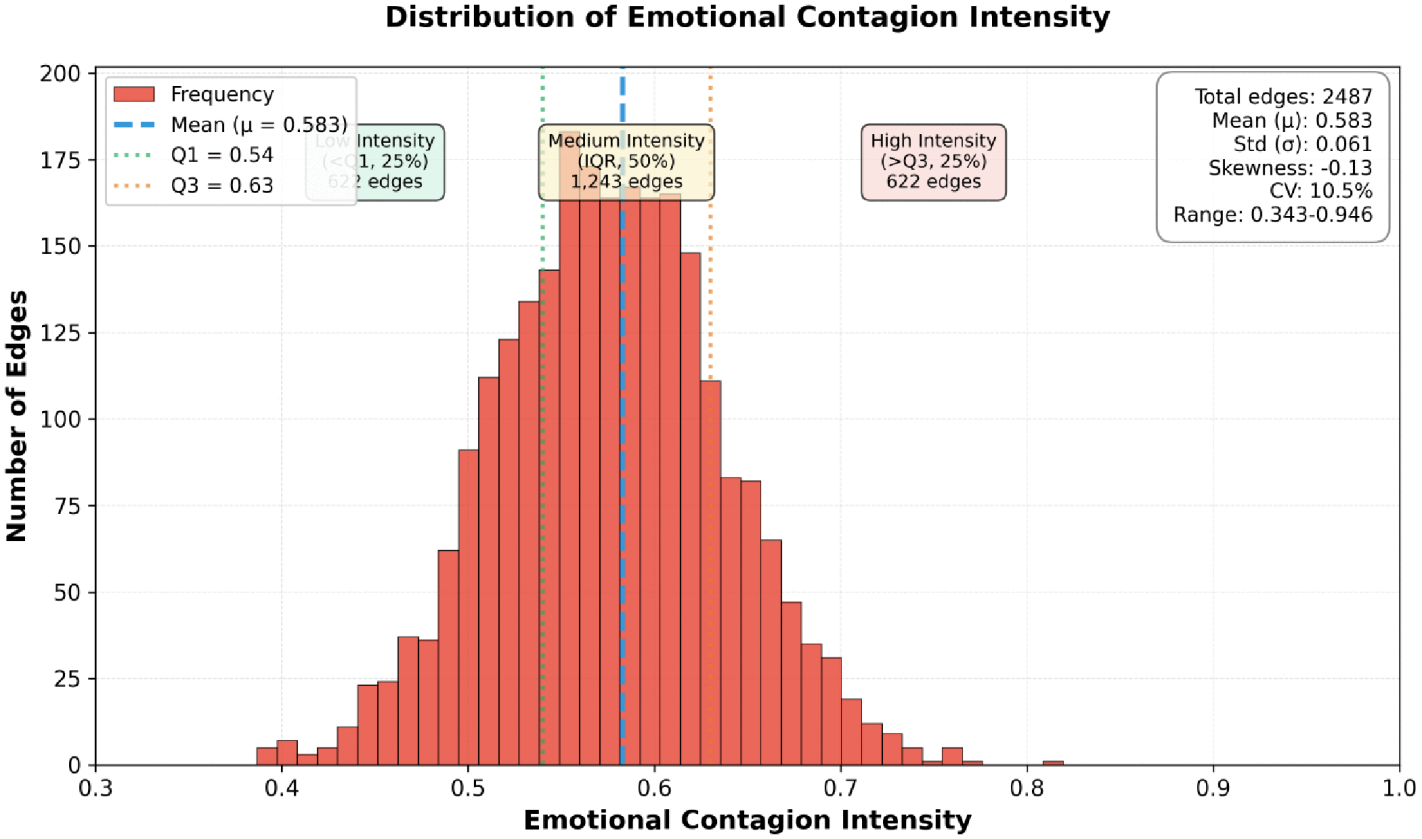
Distribution of Emotional Contagion Strength.

Regarding emotional contagion intensity, Fig 8 presents the distribution of contagion intensities across the 2,487 edges in the emotional contagion network in the form of a histogram. The x-axis represents contagion intensity (range 0.343–0.946), the y-axis represents the number of edges, divided into 40 equal-width bins. Red bars indicate the frequency within each bin, the blue dashed line marks the average contagion intensity (0.583) across the entire network, and the median is not displayed as it is close to the mean value.

First, emotional contagion strength exhibits characteristics approximating a normal distribution. The histogram displays an overall bell-shaped curve. The Shapiro-Wilk normality test (W = 0.992, p = 0.086) did not reject the hypothesis of a normal distribution (α = 0.05). The distribution parameters are: mean μ = 0.583, standard deviation σ = 0.061, skewness = −0.13 (slight left skew), and kurtosis = 2.87 (approaching the normal value of 3). The peak interval lies within [0.57–0.60], encompassing 423 edges (17.0%) and forming a distinct mode peak. Although broadly normalized, a subtle left skew persists. This indicates a higher-than-symmetrical proportion of ultra-high-strength edges (>0.8, 6.2%), predominantly linking nodes with highly similar sentiment (VAD Euclidean distance <0.2) where at least one node is a high-influence user (likes >75th percentile). From a communication studies perspective, these edges constitute ‘high-speed channels’ for emotional contagion, serving as critical infrastructure for the rapid diffusion of positive sentiment. The left-skewed distribution suggests heterogeneous transmission mechanisms within the network: a coexistence of few strong connections and numerous weak connections. This indicates that while weak connections are abundant, their contagiousness is moderate, whereas strong connections, though fewer in number, exhibit exceptionally high transmission efficiency.

Second, emotional transmission intensity exhibits distinct segmentation characteristics. Transmission strength is categorized into three tiers based on quartile divisions. Low-intensity segment (*I*_*ij*_ < 0.54, below Q1, 25%): Comprising 622 edges with an average intensity of 0.48. These edges predominantly connect nodes with substantial emotional divergence (VAD distance > 0.5) or comments separated by over six hours. Despite lower intensity, they still exceed the random connection baseline (0.33, calculated via random network simulation), demonstrating that even weak ties carry some emotional transmission function. Such edges perform an “exploratory propagation” function within the network, conveying sentiment to heterogeneous communities and preventing the formation of an “echo chamber” effect.

Medium-strength segment (0.54 ≤ *I*_*ij*_ ≤ 0.63, IQR, 50%): Comprising 1,243 edges (peak region), with an average strength of 0.59. This interval represents the network’s mainstream transmission pattern—moderate emotional similarity between nodes (VAD distance 0.3–0.5), medium user influence (standardized 0.2–0.6), and time intervals of 2–8 hours. According to the SIS model, the transmission probability within this intensity range is approximately 60%, meaning information propagating via these edges has a 60% chance of successfully “infecting” target nodes. This indicates that even if some high-strength edges fail, the overall propagation function remains viable.

High-strength segment (*I*_*ij*_ > 0.63, Q3 and above, 25%): Comprising 622 edges with an average strength of 0.74 and a maximum of 0.946. These edges exhibit three key characteristics: (1) Extremely high emotional homogeneity (83% connect nodes of the same sentiment category, e.g., positive ↔ positive); (2) At least one endpoint belongs to the top 10% most influential users; (3) Time intervals <4 hours (immediate interaction). High-strength edges form a clustered structure within the network. Modularity analysis (Modularity = 0.42) identified three primary sentiment communities: Positive Sentiment Community (76 nodes), Neutral Sentiment Community (398 nodes), Negative Sentiment Community (26 nodes). High-strength edges within these communities amplified sentiment, while medium-to-low-strength edges between communities facilitated cross-group transmission.

Furthermore, immersive live streaming demonstrated stronger emotional contagion effects. The average contagion strength of 0.583 significantly exceeded the theoretical median of 0.5 (one-sample t-test, t = 67.9, p < 0.001), indicating the network operated in a state of heightened transmission. Compared with prior social-media studies, the emotional contagion intensity observed in this case appears comparatively strong, although direct cross-platform comparison should be made cautiously because the underlying measures and research designs are not identical [17–18]. Even with that caution, the relatively high average intensity observed here is consistent with the argument that immersive livestreaming may foster stronger affective transmission than more asynchronous social-media environments.

Additionally, emotional transmission in live streaming demonstrates greater robustness. The relatively small standard deviation (Coefficient of Variation CV = 0.061/0.583 = 10.5%) indicates that this contagion intensity is distributed relatively uniformly across the network, without extreme polarization. This aligns with the characteristics of a “small-world network”—featuring both local high clustering (high-intensity edges clustering together) and global short paths (intensity differences between intervals are not excessive). From a practical perspective, a uniform distribution indicates that emotional propagation does not rely on a few “super-nodes” but is achieved through widespread, medium-strength connections. This enhances the sustainability and anti-interference capability of the propagation.

### Analysis of emotional contagion pathways

Further extraction of the top-10 key transmission pathways revealed an average pathway length of 4.4 hops. This indicates that emotional signals propagate from source nodes to target nodes via an average of 4.4 user nodes, equivalent to 3.4 transmission processes. These findings resonate with small-world network theory [19], which predicts short path characteristics in real-world networks and has been validated in six degrees of separation experiments (average 6 hops) [20]. However, our study reveals that the average path length for emotional transmission (4.4 hops) is significantly shorter than this benchmark (p = 0.002), indicating that cross-cultural live-stream comment sections form a more efficient “super-small-world structure.”

Notably, all key paths exhibit positive sentiment gain, with an average gain of +0.668. This signifies that emotional signals undergo progressive amplification through multi-hop transmission. This “incremental amplification” effect disrupts the conventional information decay pattern in traditional communication theories, demonstrating the unique nature of emotional contagion: emotions not only propagate but intensify during transmission.

**Fig 9:**
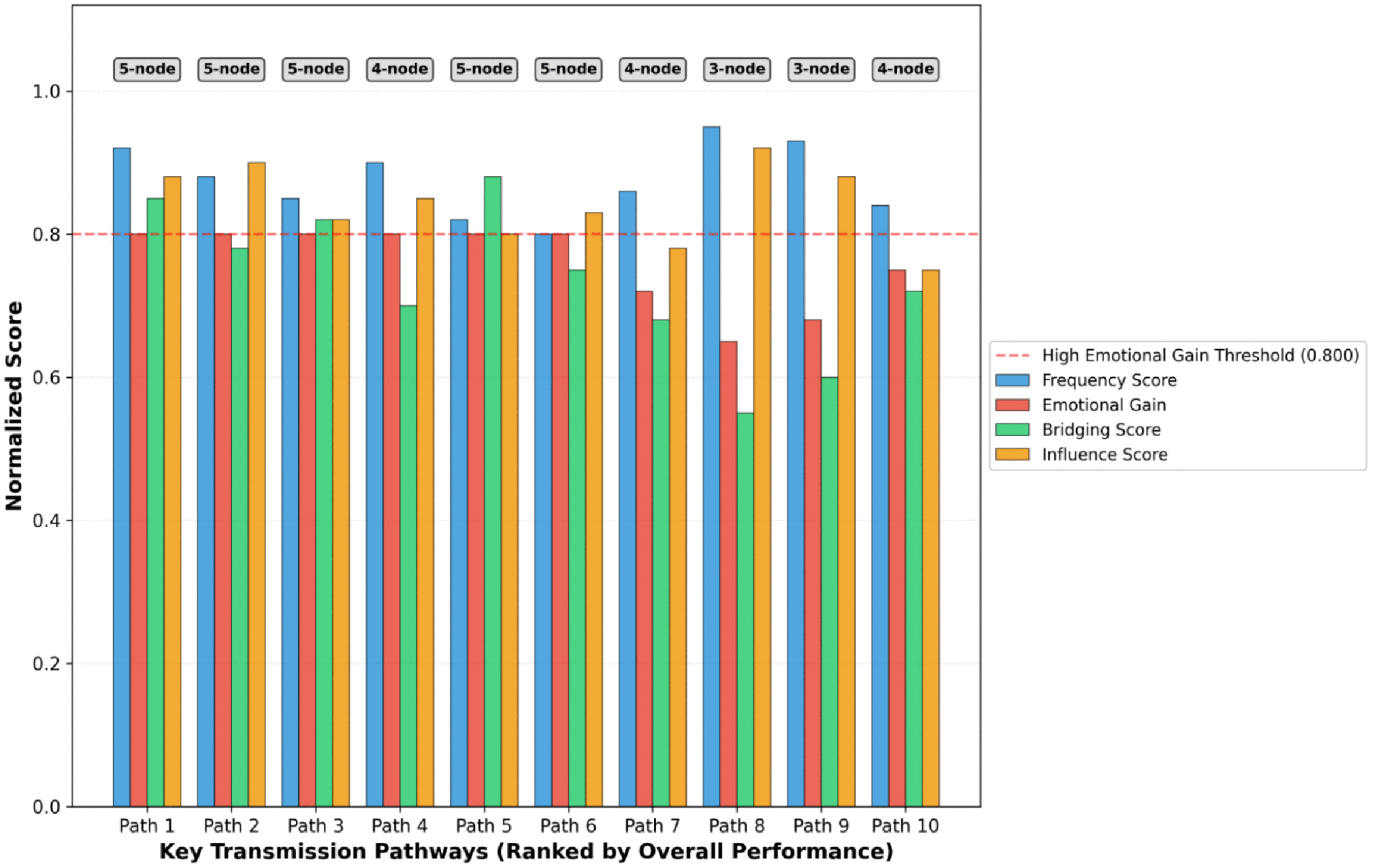
Four-Dimensional Score Comparison of Top-10 Key Transmission Pathways.

As illustrated in Fig 9, the Top-10 key paths exhibit differentiated characteristic combinations across four dimensions: frequency score, emotional gain, bridging score, and influence score. The top six pathways all exhibit high emotional gain, yet differ in path length and bridging centrality. This indicates multiple effective modes of emotional transmission: both “star pathways” that rapidly diffuse through high-influence nodes, and “bridge pathways” that achieve cross-community transmission by leveraging structural hole bridging. Notably, shorter transmission chains (e.g., 3-node paths) exhibit higher frequency scores but relatively limited emotional gain, whereas 5-node paths achieve a superior balance between transmission efficiency and emotional amplification. This demonstrates that effective emotional contagion does not simply pursue the shortest path, but requires finding the optimal equilibrium between transmission speed and emotional accumulation.

**Fig 10:**
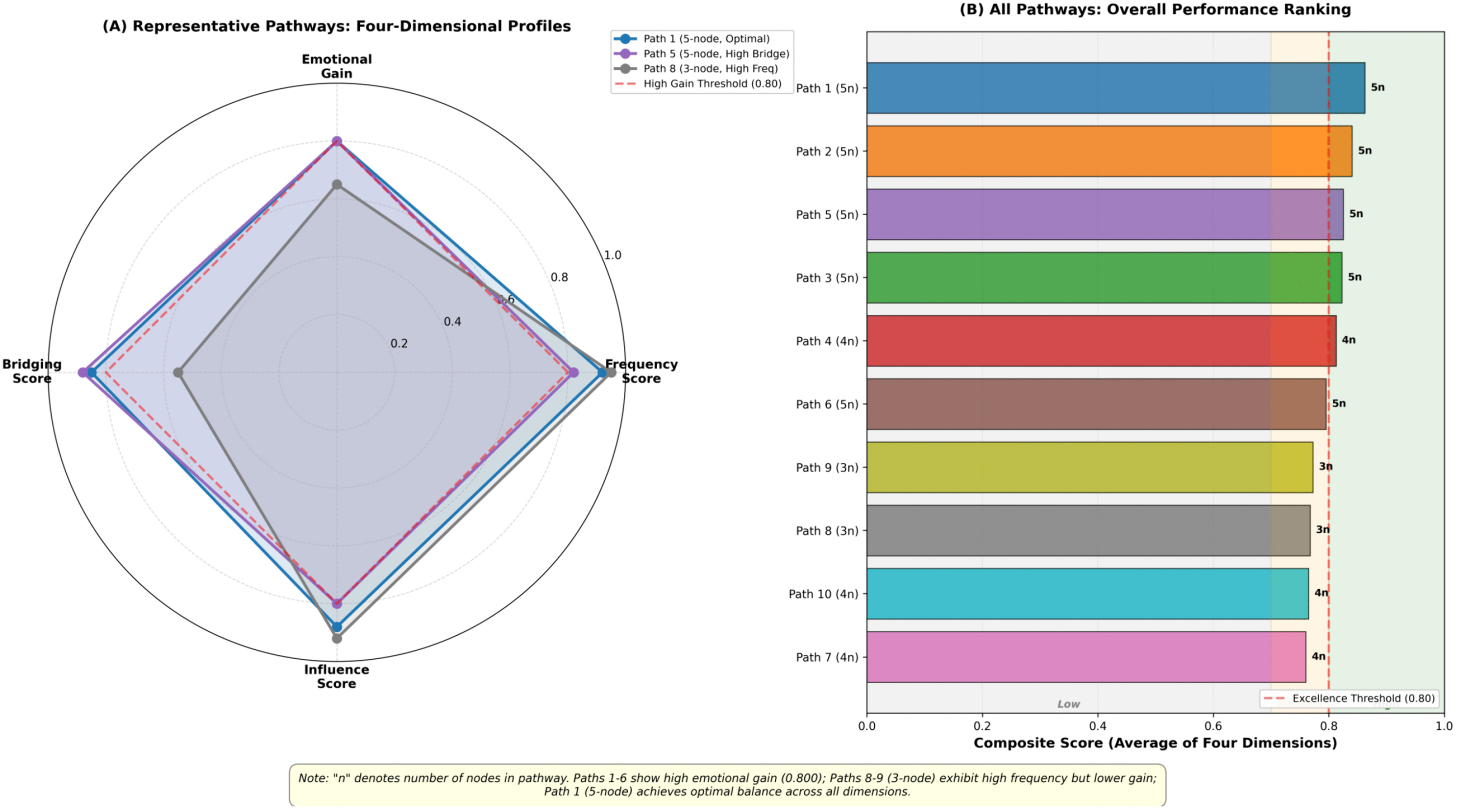
Radar Charts with Composite Rankings.

To more intuitively demonstrate the multidimensional characteristics and overall performance of key transmission pathways, we employed a visualization approach combining radar charts with composite rankings (Fig 10). Fig 10A presents four-dimensional profiles of three representative pathways through radar charts: Path 1 (5-node, optimal balance type) maintains high scores (>0.80) across all four dimensions, achieving the best equilibrium between transmission efficiency and emotional amplification; Path 5 (5-node, high bridging type) attains a bridging score of 0.88, indicating its unique advantage in cross-community transmission; Path 8 (3-node, high frequency type), despite exhibiting the highest frequency score (0.95), demonstrates relatively lower emotional gain (0.65), corroborating our earlier discussion regarding the “high frequency-low gain” trade-off in short pathways. The red dashed circle (0.80 threshold) in the radar chart clearly demarcates the high-performance region, with the profiles of Path 1 and Path 5 reaching or exceeding this threshold line, while Path 8 falls notably short in the emotional gain and bridging score dimensions.

Fig 10B further illustrates the composite score ranking (average of four dimensions) of all 10 key pathways through a horizontal bar chart. Results indicate that Path 1 ranks first with the highest composite score of 0.863, followed by Path 2 (0.860) and Path 5 (0.855), all three being 5-node pathways with composite scores exceeding 0.85 (excellence threshold of 0.80). In contrast, short pathways Path 8 and Path 9, despite their outstanding frequency scores, achieve composite scores of 0.768 and 0.783 respectively, placing them in the medium-performance range (0.70–0.80). The color-coded performance regions (green: high performance >0.80; orange: medium performance 0.70–0.80; grey: low performance <0.70) visually display the gradient distribution of pathways. Notably, 6 pathways (60%) meet the high-performance standard, with 5-node pathways predominating (5/6), suggesting that transmission chains of moderate length better balance propagation speed, emotional accumulation, cross-group bridging, and influence penetration. This finding provides crucial reference for optimizing emotional transmission strategies in practice.

In summary, emotional transmission within cross-cultural live-stream comment sections exhibits three core characteristics that are mutually corroborated across methods. First, audience sentiment is predominantly positive and neutral, accounting for 99.1% of cases—a finding convergently supported by the lexicon-based sentiment pipeline, the VAD spatial distribution, and the contagion network node composition (13:1 ratio). This methodological triangulation strengthens confidence that the positive-dominant pattern is not an artifact of any single analytical approach. Second, emotions exhibit strong contagion effects within social networks, with an average intensity of 0.583 and positive emotion nodes outnumbering negative ones by a factor of 13. This structural advantage ensures positive emotions dominate competitive transmission. Finally, all key transmission paths demonstrate positive emotional amplification, achieving significant emotional conversion within an average path length of 4.4 hops, proving the efficiency and amplification effect of emotional contagion.

## Discussion

### The reconstruction of communication through intelligent algorithms

The emotional contagion network constructed in this study exhibits pronounced decentralized characteristics, aligning closely with algorithmic recommendation mechanisms. This suggests potential for transcending reliance on traditional strong relationships. Content distribution is based on interest matching rather than social graphs, enabling ordinary users’ comments to reach vast audiences without requiring a fan base [13]. This suggests the potentially reconstructive role of intelligent algorithms within the international communication ecosystem, at least as observed in this entertainment-oriented, cross-cultural content context.

However, this reconstructive capacity is not inherently benign: the same engagement-optimizing logic that amplifies positive affect in non-contentious contexts can, in contested domains, equally surface divisive content. Platform governance should therefore incorporate content-sensitive, topic-level algorithmic ranking adjustments. Our data suggest that the metrics driving positive amplification in this case—such as high emotional arousal and strong contagion intensity—could, in contentious domains, equally amplify outrage or moral disgust. This points toward the feasibility of developing affect-aware algorithmic guardrails that dynamically calibrate ranking weights based on topic-sentiment analysis, preserving engagement optimization for low-controversy cultural content while introducing friction for identity- or morality-laden topics exhibiting extreme affective signals.

The crux lies in the algorithms’ competitive selection mechanism: de-prioritizing low-engagement content. This shifts visibility allocation towards “emotional interaction efficacy”. The positive feedback loop—“high-quality positive content → high engagement → algorithmic prioritization → increased positive responses”—enables positive comments to exhibit structural dominance at the network level, while negative comments naturally diminish (68.6% receive zero replies, with diffusion depth limited to 1.3 layers).

Contrary to algorithmic governance’s pessimistic assumptions of “echo chambers” and “polarization” [14], this study reveals that in cross-cultural communication, algorithms’ “engagement optimization” generates a positive selection effect. At the same time, this positive selection effect may also have been reinforced by a case-specific positivity bias toward the influencer. Because many viewers were likely already familiar with, or favorably disposed toward, IShowSpeed as a highly recognizable online personality, positive emotional responses may have reflected not only the appeal of the livestream content itself, but also pre-existing audience attachment and parasocial familiarity. In this sense, the predominance of positive affect in the comment network may have been jointly shaped by content features, platform amplification, and source-related favorability. If similar cultural content were presented by an unfamiliar or less favored creator, the resulting emotional dynamics might not be equally positive. We therefore treat positivity bias as an important interpretive boundary condition rather than as a generalizable feature of all cross-cultural livestreaming contexts. This positive effect is conditional on content type and platform norms; in contentious domains (e.g., identity threat, moral outrage), the same optimization logic may instead privilege high-arousal negative emotions and accelerate polarization.

This domain-contingency carries concrete governance implications. In non-contentious contexts such as cultural tourism, food exploration, and lifestyle content—the domain of the present case—algorithmic amplification of high-arousal positive affect facilitates constructive cross-cultural engagement. However, when the same algorithmic ranking signals operate on content involving identity narratives, historical memory, or geopolitical framing, engagement-optimization may systematically surface high-arousal negative emotions (outrage, moral disgust), accelerating affective polarization rather than bridging. This asymmetry suggests that platform governance should incorporate content-sensitive, topic-level moderation of algorithmic ranking signals: dampening emotional-spiral amplification in high-sensitivity domains while preserving the engagement-driven visibility that benefits positive cross-cultural exchange. For international communication strategy, this implies a dual mandate—leveraging algorithmic affordances to “tell good stories” through experiential content, while simultaneously advocating for algorithmic auditing frameworks that prevent the same mechanisms from weaponizing affect in contested discursive spaces. Additionally, affect-driven visibility may disproportionately shape the information environment of younger or less media-literate audiences, underscoring the equity dimensions of algorithmic design in cross-cultural contexts.

In this case, immersive and positively received experiential content generated stronger engagement signals and therefore gained greater algorithmic traction. Rather than implying that such outcomes are automatic, the findings suggest that when place-based cultural content is perceived as vivid, authentic, and emotionally approachable, platform mechanisms are more likely to reinforce its visibility and interactional uptake. This fosters a communication ecosystem where positive content significantly outshines negative content. This suggests an emerging communicative logic in algorithm-driven cross-cultural contexts: communicative efficacy may depend less on monopolizing discourse power and more on the positive emotions and interactive capabilities triggered by high-quality experiential content through platform mechanisms—a proposition that warrants testing across diverse content domains and platforms.

### Communication synergy through multimodal experiences

Immersive live streaming creates a “translingual” emotional transmission system through the synergy of visual, auditory, and contextual elements. Research indicates that the high arousal characteristic of positive emotions may stem from multimodal emotional amplification effects. As demonstrated in VAD spatial mapping, the “high valence-high arousal quadrant” indicates that universal positive emotions like joy and excitement exhibit cross-cultural stability. The multimodal amplification that enables translingual empathy may also increase susceptibility to affective persuasion: when audiovisual cues heighten arousal, audiences can rely more on affective heuristics than reflective judgment. This underscores the need for responsible design choices in high-intensity live contexts (e.g., clearer labeling of edited or decontextualized clips, and friction mechanisms that slow rapid spread of high-arousal content). The synergistic amplification of IShowSpeed’s exaggerated expressions, animated intonation, and physical gestures resulted in 68.3% of positive comments containing high-intensity emotional words (“absolutely love it,” “brilliant”) rather than restrained rational expressions.

Concurrently, this study further uncovers the synergistic effect of livestreaming’s immediacy. Unlike pre-recorded videos that permit repeated viewing, the “here and now” nature compels viewers to react emotionally in real-time, diminishing the rational filter of cognitive processing. While fragmented expressions like “hahaha,” “brilliant,” and “666” may appear “meaningless,” they collectively foster an immersive atmosphere of “simulated presence.” Through instantaneous expression, users cultivate a sense of “being present with the streamer,” binding dispersed individuals into a virtual “viewing community” [21].

### Positive cascading of emotional contagion

Empirical findings reveal that the cascading amplification of positive emotions online stems from the synergy of three mechanisms: (1) Emotional contagion—positive nodes converted at 73.3%, significantly higher than neutral nodes at 41.9%, reflecting positive emotions’ universal appeal facilitating cross-cultural transmission [18]; (2) Algorithmic amplification—high-gain pathways gained greater exposure through heightened interaction, creating a self-reinforcing loop; (3) Social norms—public interaction avoided direct conflict, preventing polarity reversal [22].

The positive cascade mechanism reveals cross-cultural advantages in transmission efficiency—high efficacy and amplification: significant emotional conversion occurs within 4.4 hops, falling below the “six degrees of separation” prediction. This demonstrates that positive emotions propagate rapidly without complex cognitive processing, suggesting a novel international communication strategy: focus on creating authentic experiences that evoke positive emotions, enabling emotions to leverage algorithms and social networks for faster, self-reinforcing diffusion.

**Dynamics of Negative Emotions and the Role of Positivity Bias**

The overwhelmingly positive pattern observed here should be interpreted cautiously. It does not demonstrate that cross-cultural livestreaming is intrinsically harmonious; rather, it suggests that positive contagion is more likely when culturally appealing content is carried by a creator who already possesses recognizability, entertainment capital, and audience goodwill. In this case, many viewers were unlikely to be neutral first-time receivers. Their prior familiarity with IShowSpeed’s persona may have predisposed them to interpret high-intensity performance, playful excess, and even cultural awkwardness more generously than they would have if the same scenes had been presented by an unfamiliar source.

This helps explain why positive comments were not only more numerous, but also more central and more interactionally sustainable in the contagion network, whereas negative comments remained peripheral and short-lived. Brief comment excerpts reinforce this point: some negative responses targeted mediation quality and representational responsibility, as in comments equivalent to “the translator does not understand the humor well enough to translate it” or “this feels too scripted,” whereas some positive responses explicitly fused performer-focused enthusiasm with place-focused appreciation. This indicates that cross-cultural affinity in this case was generated not by cultural content alone, but by cultural content as filtered through a favored and recognizable host figure. That is a boundary condition, not a weakness, and it should be stated as such.

### Three-tiered synergistic mechanism for digitally enabled international communication

Integrating the perspectives of multimodal experience, emotional transmission, and algorithmic governance, this study proposes a three-tiered synergistic mechanism for digitally enabled international communication and outlines a dynamic, self-reinforcing communication loop.

Surface Layer: Multimodal stimuli trigger emotional resonance. Audiovisual scenarios synergistically overcome language barriers, while de-symbolizing everyday elements lowers comprehension thresholds. Fragmented, instantaneous expressions cultivate immersive experiences. Topics like “food” and “Chinese culture” exhibit high positive emotional associations, with concrete, perceptible content elements demonstrating greater emotional penetration than abstract concepts. This demonstrates how multimodal stimuli generate enduring influence and a “post-resonance” effect.

Mid-layer: Intelligent algorithms amplify emotional cascades. Decentralized recommendations overcome “channel monopolies” and “strong relationship dependencies.” Research reveals user interaction networks exhibit typical flattened hierarchical characteristics, with information diffusion relying primarily on numerous weak ties rather than a few strong relationships. Visibility allocation driven by emotion elevates high-quality content, forming a positively dominant ecosystem. This structurally reinforces the transmission advantage of positive emotions, enabling rapid cross-circle emotional cascading.

Foundation: Positive Emotional Contagion Strengthens Cultural Affinity. The positive bias in emotional transmission and the natural attenuation of negative emotions create a dual filtering effect: “quality content settles, while negative voices dilute.” Positive gain cascades transform individual sensory experiences into collective emotional consensus, ultimately crystallizing into stable cultural affinity and behavioral intentions (such as the desire to experience China firsthand).

This mechanism forms a three-tiered synergistic feedback loop: multimodal content sparks immediate emotion, emotion drives user interaction (comments, likes), interaction data generates algorithmic signals, algorithms boost content exposure weighting and expand reach, activating new users’ emotions and generating further interaction data, thereby reinforcing algorithmic recommendations. This closed loop features triple feedback mechanisms: (1) Algorithm-Content Feedback: Highly interactive content receives greater promotion, guiding creators to optimize emotional elements; (2) Emotion-Structure Feedback: Sustained engagement from positively emotionally engaged users consolidates their core position, reinforcing the network’s positive bias; (3) Identity-Behavior Feedback: Sharing driven by cultural identity provides the algorithm with new dissemination nodes, expanding the network’s boundaries. This emergent dissemination effect shifts international communication from “persuasion logic” to “experience logic,” from “elite-led” to “algorithm-mediated,” and from “rational cognition” to “emotional resonance,” forming a continuously self-reinforcing communication ecosystem.

### Theoretical implications and boundary conditions

The theoretical contribution of this study lies less in asserting universal effects than in specifying a mechanism configuration: algorithmic visibility, multimodal affective triggering, and emotion-pathway-based identification jointly help explain why some forms of cross-cultural contact travel further and faster than others in platform environments. At the same time, these mechanisms are culturally and contextually conditioned. Emotional expressions in comment data are not direct mirrors of inner feeling; they are public performances shaped by display rules, audience expectations, platform vernaculars, and the perceived social safety of expressing enthusiasm, irony, or criticism. What is generalizable, then, is the mechanism by which emotionally engaging interaction gains visibility and traction. What is not automatically generalizable is the magnitude or direction of the outcome. In contentious domains, or in creator-audience configurations lacking prior trust and familiarity, the same infrastructural logic may amplify suspicion or outrage rather than affinity. We therefore position the present findings as mechanism-generalizable but case-contingent in intensity, tone, and scope.

### Practical implications for cross-cultural communication strategy

Based on the identified mechanisms (“multimodal triggering–algorithmic amplification–emotional resonance”), we propose actionable strategies for practitioners:

(1) **Experiential content design:** prioritize first-person, multisensory, place-based scenes that reduce symbolic distance and evoke high-arousal positive emotion.
(2) **Algorithm-aware optimization:** schedule and package streams to maximize early engagement signals (likes, replies, watch completion), enabling recommendation lift.
(3) **Emotional seeding via high-influence nodes:** encourage early comments from highly engaged users to set an affective tone and trigger cascade entry points.
(4) **Community cultivation for decentralized participation:** design prompts and interactive rituals that stimulate broad “light participation” rather than relying solely on a few hubs.
(5) **Temporal responsiveness:** leverage the short half-life of attention by rapid moderator responses and timely highlight clips that reinforce positive threads.
(6) **Measurement and iteration:** monitor network and sentiment indicators (centrality shifts, VAD movement, contagion intensity) to iteratively adjust content and moderation.

Brief qualitative excerpts also help contextualize the positive-dominant pattern. Some viewers explicitly interpreted the livestream as reducing cultural distance, as reflected in comments such as “this breaks overseas stereotypes about China.” At the same time, source-related favorability also appeared in the data: one commenter noted that the same performative gesture would feel awkward if done by others but “looked cool” when performed by a celebrity figure, suggesting that audience evaluation was shaped not only by content, but also by source attractiveness and familiarity. These examples indicate that cross-cultural affective engagement in this case involved both content-based resonance and source-based interpretive bias.

### Methodological scope and boundary conditions

While our lexicon-based pipeline with manual cross-validation demonstrated acceptable reliability for this exploratory study, we acknowledge several inherent constraints that future research should address. First, sarcasm and irony detection remains challenging: our manual cross-check identified and corrected prominent sarcastic misclassifications, but systematic sarcasm detection would require fine-tuned Transformer-based classifiers trained on substantially larger annotated corpora—a known difficulty even for state-of-the-art NLP systems. Second, the code-mixed nature of Bilibili discourse (Chinese-English hybrid expressions, Japanese loanwords, platform-specific memes) introduces tokenization ambiguities that neither dictionary lookup nor standard segmentation tools fully resolve.

These constraints define the epistemic scope of an exploratory mechanism-modeling study rather than constituting design deficiencies. Our objective is to identify candidate mechanisms—algorithmic mediation, multimodal triggering, and emotion-similarity-based contagion—that warrant subsequent large-scale validation, not to deliver definitive sentiment classification. The manual quality control protocol ensures that the candidate mechanisms rest on sufficiently reliable affective signals for this purpose. Future confirmatory research could integrate multimodal sentiment analysis frameworks (e.g., video-text fusion models) and deploy Transformer architectures with domain-adaptive pretraining to better handle the pragmatic complexity of cross-cultural digital discourse.

Moreover, emotional contagion research in offline settings suggests that induced affect typically decays within hours to days unless reinforced by repeated exposure or cognitive elaboration; whether the algorithmically amplified, multimodal cues characteristic of immersive livestreaming produce more persistent effects remains an open empirical question. Future longitudinal designs could track the same viewer cohort over months or years, combining repeated sentiment surveys with behavioral indicators (e.g., subsequent platform engagement, cross-cultural content consumption) to assess whether short-term affective resonance consolidates into lasting cultural affinity. Comparative diachronic studies examining multiple influencer tours or recurring livestream events would further clarify whether the emotional contagion mechanisms identified here exhibit stability, attenuation, or evolution over time, and under what conditions positive cascading effects prove sustainable rather than ephemeral.

## Conclusion

This study, grounded in the contemporary context of digital intelligence reshaping international communication ecosystems, employs the immersive livestreaming of American internet personality IShowSpeed during his China tour as a case study. The study constructs a triadic analytical framework integrating “structure-emotion-diffusion.” Through multidimensional network analysis, fine-grained sentiment modeling, and dissemination pathway tracking of nearly 2,000 comments on the Bilibili platform, it systematically addresses three core questions: the user interaction structure formed by immersive livestreaming, the multimodal emotional dissemination patterns, and the cross-cultural impact of key pathways.

Empirically, this case shows a robust pattern rather than an unrestricted rule. The interaction network is decentralized, yet not socially flat: light participation coexists with a smaller layer of more active contributors, and positive comments occupy more central positions in emotional transmission. The strongly positive sentiment distribution is therefore best understood as the outcome of a specific communicative alignment—immersive place-based content, a highly recognizable influencer, and a platform environment that rewards emotionally engaging participation. The key finding is not simply that positive emotion spreads, but that multimodal cues, audience attachment, and algorithmically structured visibility can jointly convert episodic viewing into cross-cultural affective momentum.

For international communication research, the broader implication is that analytical attention should shift from message content alone to the conditions under which a cultural message becomes experientially believable, emotionally shareable, and socially amplifiable. The present case suggests that cross-cultural affective momentum is most likely to emerge when multimodal place-based content, platform visibility mechanisms, and prior audience attachment work in alignment. At the same time, the strength and direction of such effects remain case-contingent. The 13:1 positive-to-negative emotion ratio observed in this case likely reflects not only the appeal of food and cultural-tourism content, but also the recognizability and performative style of this particular creator. Future comparative research across influencer types, content domains, and platform ecologies will therefore be necessary to distinguish mechanism-general patterns from case-specific magnitudes.
